# Evaluation of the involvement of Th17-cells in the pathogenesis of canine spinal cord injury

**DOI:** 10.1371/journal.pone.0257442

**Published:** 2021-09-30

**Authors:** Annika Kämpe, Anna Knebel, Regina Carlson, Karl Rohn, Andrea Tipold

**Affiliations:** 1 Department of Small Animal Medicine and Surgery, University of Veterinary Medicine, Hannover, Germany; 2 Department of Biometry, Epidemiology and Information Processing, University of Veterinary Medicine Hannover, Hannover, Germany; Szegedi Tudomanyegyetem, HUNGARY

## Abstract

Intervertebral disc herniation (IVDH) is a frequently occurring neurological disease of dogs and the most common reason for spinal cord injury (SCI). Clinical signs are variable thus a reliable prognosis is crucial for further treatment decisions. Currently, the prognosis of IVDH primarily depends on presence or absence of deep pain perception. The purpose of this study was to investigate if Th17-cells could serve as a potential, prognostic biomarker for IVDH. We investigated a possible role of the adaptive immune system in the pathophysiology of IVDH in dogs. The investigation was performed by analyzing the influence of Th17-cells in blood and cerebrospinal fluid (CSF) of sixty-two dogs suffering from IVDH. In addition, we examined if Th17-cells might influence the course of this disease. As controls, paired blood and CSF samples of ten healthy clinic-owned dogs were examined and the values were compared to those of the IVDH group. Isolated lymphocytes were analyzed after stimulation by using multicolour flow cytometry to measure the number of Th17-cells. IL-17 levels were measured in paired serum and CSF samples by Enzyme‐linked Immunosorbent Assays (ELISA). Highly significant differences of stimulated Th17-cells in EDTA-blood samples could be determined between Th17-cell levels of dogs suffering from IVDH and the healthy control group and also between three sampling time points: preoperative, after clinical improvement and after six months. Preoperatively, Th17-cell levels were strongly decreased in contrast to the healthy controls. The decreased amount of Th17-cell levels recovered postoperatively so that Th17-cell levels of the last follow-up examinations were comparable to the control group after six months. At the same time IL-17 measured in serum preoperatively was significantly higher in dogs with IVDH than in healthy controls. However, there was no considerable difference of IL-17 measured in CSF between the groups. In conclusion, a high activity and consequent consumption of IL-17-producing Th17-cells is suspected in acute IVDH. These findings may indicate an involvement of Th17-cells in the pathogenesis of IVDH and emphasize that these cells might be involved in the interaction of pain, stress and immune reaction. However, based on the findings of this study the development of Th17-cells as a biomarker cannot be recommended, yet.

## Introduction

The most common cause for spinal cord injury (SCI) in dogs is intervertebral disc herniation (IVDH) associated with variable neurological deficits of different severity [1–3]. Clinical signs vary from hyperesthesia to paresis and plegia accompanied with loss of deep pain perception [2,3]. In most cases further, detailed diagnostics are needed to evaluate the severity of a disc herniation [4]. International gold standard for clinical diagnosis is the magnetic resonance imaging (MRI) [4,5].

While dogs with an intact deep pain perception generally regain mobility after appropriate treatment, a reliable prognosis, particularly for dogs without deep pain perception is difficult [6,7].

Objective parameters which might contribute to providing a reliable prognosis and supporting subsequent therapy are still not available [8]. To date the prognosis is mainly based on the presence or absence of deep pain perception [7]. WANG-LEANDRO et al. compared several methods in order to predict prognosis and found the presence of deep pain perception within paraplegic dogs a more reliable forecast for functional rehabilitation compared to measurable parameters based on magnetic resonance imaging data [9]. An additional option for assessing the prognosis is the classification of the severity grades based on SHARP and WHEELER (2005) [3]. According to SHARP and WHEELER (2005) plegic dogs with the loss of deep pain perception are assigned the highest severity grade and thus the worst prognosis [3].

However, several studies revealed that 52.1%–78% of paraplegic dogs without deep pain perception may regain mobility after treatment [7,10–14]. Thus, assessing a reliable prognosis is not possible and emphasizes the need of reliable parameters/biomarkers allowing a more accurate prognosis [8].

A suitable biomarker for investigation needs to be easily measurable [15]. In this context especially measurable parameters within blood, cerebrospinal fluid (CSF) and other biological materials are interesting, especially if those parameters are easily and quickly accessible [8,15].

Th17-cells, a special type of T-Helper cells, could represent such a biomarker [8,16]. Human studies also confirmed an immunopathological involvement of Th17-cells in the pathogenesis of human IVDH [17].

Interleukin-17 (IL-17)-producing helper T-cells (Th17-cells), a subgroup of CD4+ cells, which are part of the adaptive immune system, play a central role in protection against infections and in autoimmune diseases such as human psoriasis, multiple sclerosis (MS), inflammatory bowel disease (IBD), Crohn’s disease, asthma, rheumatic arthritis and ulcerative colitis [18–22]. However, the involvement of Th17-cells concerning these diseases is not completely understood until now and is also discussed in the pathogenesis of pain and secondary inflammatory reactions. FREUNDT-REVILLA et al. confirmed the hypothesis of a Th17-skewed immune response in Steroid-Responsive Meningitis-Arteritis (SRMA) in dogs [23]. Control groups included dogs with IVDH (n = 7) which showed increased levels of IFN-γ and IL-17 spot forming cells in blood in contrast to healthy controls [23]. Thus, cytokines seem to be involved in the pathogenesis of canine SCI. In addition, SPITZBARTH et al. found an upregulation of different proinflammatory cytokines (IL-6, IL-8, TNF-, TGF-ß) in dogs with acute SCI [24].

Recent studies in human patients suggest that the origin of pain related to disc herniation may be a result of an immune response or inflammation [17].

The immune system might recognize the leaking fluid of nucleus pulposus as foreign material, thus causing inflammation [17]. CHENG et al. identified increased TH17 and IL-17 levels especially in patients with intervertebral disc extrusions [25].

Since dogs proved to be excellent animal models for human diseases [24,26], it is reasonable to assume that Th17-cells are also involved in the pathogenesis of IVDH in dogs [17]. KOL et al. demonstrated that Th17-cells are measurable in peripheral blood of dogs [27]. We modified and improved the protocol of KOL et al. in order to implement a more practicable identification method during daily routine [27–29].

The aim of this study was to prove the hypothesis that Th17-cells are involved in the pathogenesis of canine spinal cord injury, these cells are useful biomarkers for the prognosis of the disease and might have an effect on the course of the disease. Therefore, we analyzed Th17-cells in blood and IL-17 levels in serum and cerebrospinal fluid (CSF) of sixty-two dogs suffering from IVDH and compared the results with the clinical data and with results of a healthy control group.

## Material and methods

### Study population

Sixty-two client-owned dogs with IVDH entrusted to the Department of Small Animal Medicine and Surgery, University of Veterinary Medicine Hannover, Germany from January 2018 to July 2018 fulfilled all inclusion criteria. The study was conducted and approved in accordance with the guidelines of the Animal Care Committee of the Government of Lower Saxony and national regulations for animal welfare (animal experiment number: 33.8-42502-05-18A290). Dogs with other potential Th17-cell associated diseases were excluded from the study [18–23].

All dogs underwent a general physical and neurological examination, complete blood cell count and radiographs of the vertebral column. After the neurological examination all dogs were classified according to the severity of their neurological deficits referring to the grading system of SHARP and WHEELER [3]:

Grade 1: spinal hyperesthesia without neurological deficits
Grade 2: ambulatory paresis with mild neurological deficits
Grade 3: non-ambulatory paresis
Grade 4: plegia with deep pain perception
Grade 5: plegia with loss of deep pain perception

Afterwards, magnetic resonance imaging (MRI; Philips Medical Systems, 3.0 Tesla, Netherlands) examination was performed in order to identify the exact localization and dimension of spinal cord injury prior to decompressive surgery. If possible, cerebrospinal fluid (CSF) was taken from cisterna magna during anesthesia prior to decompressive surgery. Decompressive surgery comprised standard hemilaminectomy [3]. After identifying the lesion site and removing articular processes the hemilaminectomy was performed using a surgical drill. To avoid spinal cord damage removal of extruded disc material is performed with care using a small curved blunt probe. With three layers the incision is closed [3].

Within the next days after surgery the neurological condition was controlled on a daily basis. At the time of improvement of the dog’s condition by one severity grade according to the grading system of SHARP and WHEELER the first follow-up test including blood sampling and measurement of Th17-cells was performed [3]. Second follow up was performed six months later during clinical controls including general and neurological examination, blood testing and measurement of Th17-cell levels. Alternatively, telephone interviews with or without laboratory testing were conducted.

Ten samples of EDTA-blood, serum and CSF of healthy clinic-owned beagles served as controls (animal experiment number: 33.8-42502-05-18A290).

For Th17-cell measurement approximately 3 ml of EDTA-blood was obtained from sixty-two dogs suffering from IVDH at three different sampling time points: preoperatively (n = 62), after clinical improvement (n = 55) and at controls after six months (n = 20). Additionally, IL-17 in serum (n = 23) and IL-17 in paired serum (n = 17) and CSF samples (n = 17) was measured.

All results were compared with the data of the healthy control group (n = 10).

### EDTA-blood samples: Th17-cell measurement by flow cytometry

3 ml of peripheral blood was required for measurement of Th17-cells. Blood was collected in sterile 5 ml EDTA-tubes on all three sampling time points.

#### Peripheral blood mononuclear cells (PBMCs) isolation

Due to the fact that Th17-cells represent a very small cell population not less than 1 – 3 ml of whole blood was needed. In addition, Th17-cells had to be purified from other cells and the Th17-cells had to be stimulated prior to flow cytometry measurements [27].

Peripheral blood mononuclear cells (PBMCs) were separated according to the manufacturer’s instructions by PBMC 24+ Spin Medium (density: 1,072 g/ml; pluriSelect Life Science, Leipzig, Germany) density gradient centrifugation, which is a commercialized separation medium. Volume of medium (PBMC 24+ Spin Medium (density: 1,072 g/ml); pluriSelect Life Science, Leipzig, Germany) and buffer (0.01 mol/L phosphate buffered saline (PBS); pH = 7.4)) depended on the volume of blood. Double amount of buffer (2:1) and the same quantity of density gradient medium as blood volume were used. After centrifugation, PBMCs were harvested from the interface between plasma and density gradient medium. To remove erythrocytes, a hypotone lysis was performed by using distilled water followed by adding double concentrated PBS (in equal volumes). For vital cell count, Trypan-Blue (Sigma-Aldrich®, Germany) was used for determining dead cells. Afterwards, antibodies and buffer concentrations for further examinations could be calculated.

#### Removal of redundant cells

Separation of Th17-cells was performed by using a modified protocol by KOL et al. [27–29]. PBMCs were blocked with Human TruStain FcX™ (BioLegend®, California, USA) and subsequently marked with the following antibodies: mouse anti dog CD8 alpha (1:5 diluted with staining buffer), mouse anti dog CD11b (1:11 diluted with staining buffer), mouse anti canine CD21 (first dilution: 1:5 with staining buffer, second dilution: 1:11 with staining buffer) and goat anti-mouse IgG microBeads (1:5 diluted with staining buffer; MACS Miltenyi Biotec, Germany). Afterwards, the cell suspension was separated using the “Deplete” program of autoMACS® Pro Separator (Miltenyi Biotec GmbH, Germany). Thus, undesired cells (CD8 alpha+, CD11b+, CD21+) were sorted out by magnetic columns and the target population of CD3+ and CD4+ cells could be assembled. All antibodies were purchased from Bio-Rad Laboratories, Inc., California, USA.

#### Th17-cell culture

The purified cell suspension containing CD3+ and CD4+ cells was resuspended in a specific lymphocyte medium (RPMI Medium1640 (Gibco™ life technologies limited, UK) with 5% FBS (fetal bovine serum; CytoGen GmbH, Germany), 1% HEPES solution (1M; Sigma-Aldrich®, Germany) and 1% PenStrep (100 U/ml Penicilin-G and 100 μg/ml Streptomycin; Sigma-Aldrich®, Germany) and divided in two wells of a 96-well-plate (BRAND plates® cellGrade™; Germany). For regeneration, the cells were placed into an incubator (37°C, 5% CO_2;_ Type INDUCELL 55, MMM Medcenter, Germany) overnight.

#### Stimulation of Th17-cells

In order to make Th17-cells better measurable for flow cytometry, the cells had to be stimulated [27]. One microtiter plate well with cells was stimulated while the other microtiter plate well remained unstimulated as control. For the stimulated cells the following stimulation medium was used: specific lymphocyte medium (as previously described) including PMA (Phorbol-12-myristat-13-acetat (25 ng/ml; Antibodies-online, Germany) and Ionomycin calcium salt (500 ng/ml; Sigma-Aldrich®, Germany). After incubation time (37°C, 5% CO_2_) of three hours Brefeldin A (1 μg/ml; Sigma-Aldrich®, Germany) was added to both microtiter plate wells. After another three hours of incubation all cells were dyed with Viobility™405/520 Fixable Dye (1:100 diluted with PBS; Miltenyi Biotec GmbH, Germany). Afterwards, the cell suspensions were split again so that three microtiter plate wells (native control, isotype control, test) were available of unstimulated and stimulated cells. Except for the native control, all other cells were dyed with 5 μl of mouse anti dog CD3:FITC (BioRad Laboratories, Inc. California, USA) and 5 μl of anti-canine CD4 PE-Cyanine7 (eBioscience, San Diego, California, USA). For cell fixation a flow cytometry fixation buffer (R&D Systems®, Minneapolis, USA) was used. Afterwards, the intracellular dyeing was performed by using Biotin mouse IgG1, k isotype Ctrl (eBioscience, San Diego, California, USA) for isotype control and IL17A-Biotin (Clone: 403D100.01/mAb5 Biotin conjugated; Dendritics SAS, France) for test samples. APC Streptavidin (BioLegend®, California, USA) was used as fluorescent label for isotype control and test cells.

For washing steps PBS buffer was used at first and after Viobility™ 405/520 Fixable Dyes (Miltenyi Biotec GmbH, Germany) coloration staining buffer was applied. After fixation all washing steps were performed by using saponine buffer (0.03% saponine solution in FACS-staining-buffer; pH = 8.0).

For flow cytometry measurement all cells were resuspended in 200 μl staining buffer.

Fluorescence of the dyed cells was detected by using a multicolor flow cytometer (MACSQuant® Analzyer 10; Miltenyi Biotec GmbH, Germany). For data analysis MACSQuantify™ Software (Miltenyi Biotec GmbH, Germany) was used.

To include potential blood parameter changes like lymphopenia or lymphocytosis the absolute cell count of Th17-cells was calculated and used for comparisons. All used antibodies and solutions are listed in the table below (Table 1).

**Table 1:** Antibodies, solutions and buffer used for Th17-cell measurement by flow cytometry.

Product name	company	order number	dilution
anti-Canine CD4 PE-Cyanine7 (Clone: YKIX302.9)	eBioscience, San Diego, California, USA	25-5040-42	1: 100 with staining buffer
APC Streptavidin (Allophycocyanin-Streptavidin)	eBioscience, San Diego, California, USA	405207	1: 200 with saponine buffer
Biotin mouse IgG1, k isotype Ctrl (Clone: MOPC-21) (1:100)	BioLegend®, San Diego, Kalifornien, USA	400104	1: 100 with saponine buffer
Brefeldin A	Sigma-Aldrich®, Germany	B5936	1 μg/ml
Fetal bovine serum (FBS)	Gibco®, Thermo Fisher Scientific, Carlsbad California, USA	10270–106	-
Flow Cytometry Fixation Buffer (1X)	R&D Systems®, Minneapolis, USA	FC004	-
Goat Anti-Mouse IgG MicroBeads	MACS Miltenyi Biotec GmbH, Bergisch Gladbach, Germany	130-048-042	1: 5 with staining buffer
Hepes solution (4-(2-Hydroxyethyl)piperazine-1-ethanesulfonic acid, 1M in H_2_O, pH = 7.0 - 7.6;)	Sigma-Aldrich®, Germany	7365-45-9	-
Human TruStain FcX^TM^	BioLegend®, San Diego, California, USA	422302	1: 20 with cell suspension
IL17A-Biotin (Clone: 403D100.01/mAb5 Biotin conjugated) Mouse monoclonal antibody	Dendritics SAS, France	DDX0330B-100	1: 100 with saponine buffer
Ionomycin calcium salt	Sigma-Aldrich®, Germany	I3909	500 ng/ml
Mouse anti dog CD11b (clone: CA16.E10)	Bio-Rad Laboratories, Inc., California, USA	MCA1777S	1: 11 with staining buffer
Mouse anti dog CD3:FITC	Bio-Rad Laboratories, Inc., California, USA	MCA1774F	1: 11 with staining buffer
Mouse anti dog CD8 alpha	Bio-Rad Laboratories, Inc., California, USA	MCA1999S	1: 5 with staining buffer
Mouse anti Canine CD21	Bio-Rad Laboratories, Inc., California, USA	MCA17881E	First dilution: 1: 5 with staining buffer Second dilution: 1: 11 with staining buffer
PBMC 24+ Spin Medium® (Density: 1,072 g/ml)	pluriSelect Life Science–Worldwide, Leipzig, Germany	60-00093-12	-
PenStrep (penicillin (G-)–streptomycin (10.000 U/ml))	Sigma-Aldrich®, Germany	P 0781	-
PMA (phorbol 12-myristate 13-acetate) 0.5mg/ml in DMSO	Antibodies-online GmbH, Aachen, Germany	100008014	25 ng/ml
RPMI medium 1640 + L-glutamine + phenolred	Gibco™ life technologies limited, UK	21875091	-
Trypan-blue	Sigma-Aldrich®, Germany	T-6146	0.4 % in PBS
Viobility™ 405/520 Fixable Dyes	Miltenyi Biotec GmbH, Bergisch Gladbach, Germany	120-028-574	1: 100 with PBS buffer

(PBS = phosphate buffered saline (PBS); pH = 7.4); saponine buffer (0.03% saponine solution in FACS-staining-buffer; pH = 8.0)).

To investigate stimulated and unstimulated Th 17-cells, cells belonging to the same blood sample were divided into two parts. One share of the cells was stimulated, and the other part was left unstimulated. Stimulated Th17-cells were much better analyzable and presentable by multicolor flow cytometry than unstimulated Th17-cells. For better presentability reasons, the following presentation of the results only includes the measured values of the stimulated Th17-cells. Also, for readability reasons the “stimulated Th17-cells” will just be referred to as “Th17-cells”, hereafter.

### IL-17 measurement by ELISA

#### Serum

For measurement of IL-17 1 ml serum was needed and obtained during blood sampling for Th17-cell measurement, preoperatively. Subsequently, serum samples were centrifuged for 10 min at 2000 x g and supernatant was frozen at -20°C until further measurements.

#### Cerebrospinal fluid (CSF)

0.5 ml CSF was taken from cerebellomedullary cistern with a sterile spinal needle (22-gauge 1.50 IN, Becton Dickinson, Madrid, Spain) under general anesthesia, after MRI and prior to decompressive surgery. CSF could be acquired in 17/62 dogs and in ten healthy control beagles. Routine CSF examination (red blood cell count, white blood cell count, glucose, albumin and total protein concentration) was performed in all CSF samples immediately after acquisition [30,31]. Afterwards, all samples were centrifuged for 10 minutes at 2000 x g and the supernatant was stored in polypropylene tubes at -20°C for further measurements.

#### ELISA

The concentration of IL-17 in all serum and CSF samples of IVDH and healthy patients was measured simultaneously by using an Enzyme-Linked Immunosorbent Assay (ELISA) kit (SEA063Ca, Enzyme-linked Immunosorbent Assay Kit For Interleukin-17 (IL-17), Cloud-Clone Corp., Houston, USA), following the manufacturer’s instructions. This kit consists of a sandwich enzyme immunoassay and is validated for in vitro quantitative measurement of IL-17 in different canine fluids according to the manufacturer (Cloud-Clone Corp., Houston, USA). All samples were measured in duplicates and the mean concentration was calculated.

The provided detection range was 1.56 – 100 pg/ml. The standard curve concentrations were 100 pg/ml, 50 pg/ml, 25 pg/ml,12.5 pg/ml, 6.25 pg/ml, 3.12 pg/ml and 1.56 pg/ml. If the concentration of IL-17 in serum/CSF was above the detection limit, samples were diluted with 0.01 mol/l phosphate buffered saline (PBS; pH = 7.4). Samples, which were repeatedly not measurable, were excluded from the study.

For spectro-photometrical measurements a Synergy™ 2 Multi-Detection Microplate Reader (BioTek® Instruments GmbH, Germany) was used and data was evaluated by Gen5™ Microplate Reader and Imager Software (BioTek® Instruments, Inc., USA).

#### Statistical analysis

All results of flow cytometry were determined with MACSQuantify Software (MACS, Miltenyi Biotec, Germany) and documented with Microsoft® Excel 2010 (Microsoft Corporation, USA).

Statistical analysis was performed with statistical software SAS, version 9.4 (SAS Institute, Cary, NC, USA), using SAS® Enterprise Guide 7.1. All quantitative parameters were tested for normal distribution (Kolmogorov-Smirnov-Test) and visual assessment of qq-plots of model-residuals. Effects of two group-levels (sick and healthy), three timepoints (preoperatively, after clinical improvement and at controls after six months) and two levels of stimulation (stimulated vs. not stimulated) to Th17-cell levels were analyzed by three-way analyses of variance (ANOVA) with both measurement times and stimulation vs. non-stimulation as repeated measurements and independent measurements between groups, taking into account interactions between the effects. Post-hoc tukey test for pairwise comparisons was calculated. Due to non-normal distribution for IL-17 in CSF, Kruskal-Wallis-Test and Wilcoxon’s two sample-Test for independent samples was performed as well as Wilcoxon’s signed rank test was used for paired observations (Time points). Potential associations and correlations between Th17-cell levels and IL-17 levels were tested by using Pearson and Spearman correlation analysis. The level of statistical significance was defined as p < 0.05.

Graphs were generated with GraphPad Prism® (Version 5.0. Fa. GraphPad Software, Inc., La Jolla, CA, USA), SAS® Enterprise Guide 7.1. (SAS Institute, Cary, NC, USA) and accordingly with Microsoft® Excel 2010 (Microsoft Corporation, Redmond, USA).

## Results

### Study population

Sixty-two dogs, 40 males (64.5%; 32.2% intact and 32.2% neutered male dogs) and 22 females (35.5%; 12.9% intact bitches, 22.6% spayed bitches), suffering from IVDH fulfilled all inclusion criteria for this study. Forty-nine dogs showed acute to subacute clinical signs (< 24 hours– 23 days) and thirteen dogs showed chronical neurological deficits (> 24 days). The median age was seven years (range: 2 – 13 years) and the median bodyweight was 10.95 kg (range: 2.6–51.8 kg).

Fifteen breeds were represented. The most commonly affected breeds were dachshund (n = 12; 19.4%) and french bulldog (n = 12; 19.4%), followed by mix breeds (n = 9; 15%), jack russell terrier (n = 6; 9.7%), beagle (n = 4; 6.5%) and two dogs (3.2%) of each of the following breeds: dalmatian, yorkshire terrier as well as one dog (1.6%) of each of these breeds: hanoverian bloodhound, maltese, pug dog, rottweiler, spaniel, welsh corgi pembroke and miniature schnauzer.

The majority of dogs (18/62) were classified as grade II, and grade III (22/62). The other patients were allocated between the grade I (9/62), grade VI (8/62) and grade V (5/62) according to the grading system of SHARP and WHEELER [3].

43.5% of the dogs (27/62) showed ambulatory paresis at initial evaluation and 56.5% of the dogs (35/62) were not able to walk independently.

In 55 of 62 dogs MRI examinations (MRI; Phillips Medical Systems, 3.0 Tesla, Netherlands) were performed to confirm the suspected localization and the dimension of the lesion within the spinal cord. Three of 62 dogs were euthanized at the time of diagnosis without any treatment attempt due to the severity of clinical signs and expected poor prognosis, at the owner’s request. Accordingly, 59/62 dogs had been treated of which 51/59 dogs underwent decompressive surgery and 8/59 dogs were treated conservatively. Successful treatment occurred in 88.1% of dogs (52/59), 6.8% (4/59) did not improve clinically and 5.1% (3/59) deteriorated. These three dogs were euthanized in the first week after surgery because of deterioration of the neurological condition. Two further dogs were euthanized before the last follow-up because of other reasons than neurological diseases (68 day – 5 months). Overall, 8/62 dogs were euthanized during the study period.

Of euthanized dogs the date, cause of death, and the latest recorded neurological status were recorded.

Six months outcome data was available for 46/54 dogs. Eight dog owners could not be contacted and were lost to follow-up. These patients were excluded from statistical analysis (six months follow up).

Ten values for each of the tested parameters were obtained from healthy dogs, which served as a control group. The control group was composed of fifteen healthy, clinic owned beagles with a median age of two years (range: 1 – 5 years) and a median weight of 13.4 kg (range: 10.2–16.5 kg). The healthy control group included ten intact male dogs and five intact bitches.

### Comparison of unstimulated and stimulated Th17-cells by using flow cytometry

Since Th17-cells physiologically represent a fairly small cell population in blood, it is necessary to stimulate these cells to be able to detect significant differences between groups [23,27].

**Fig 1:**
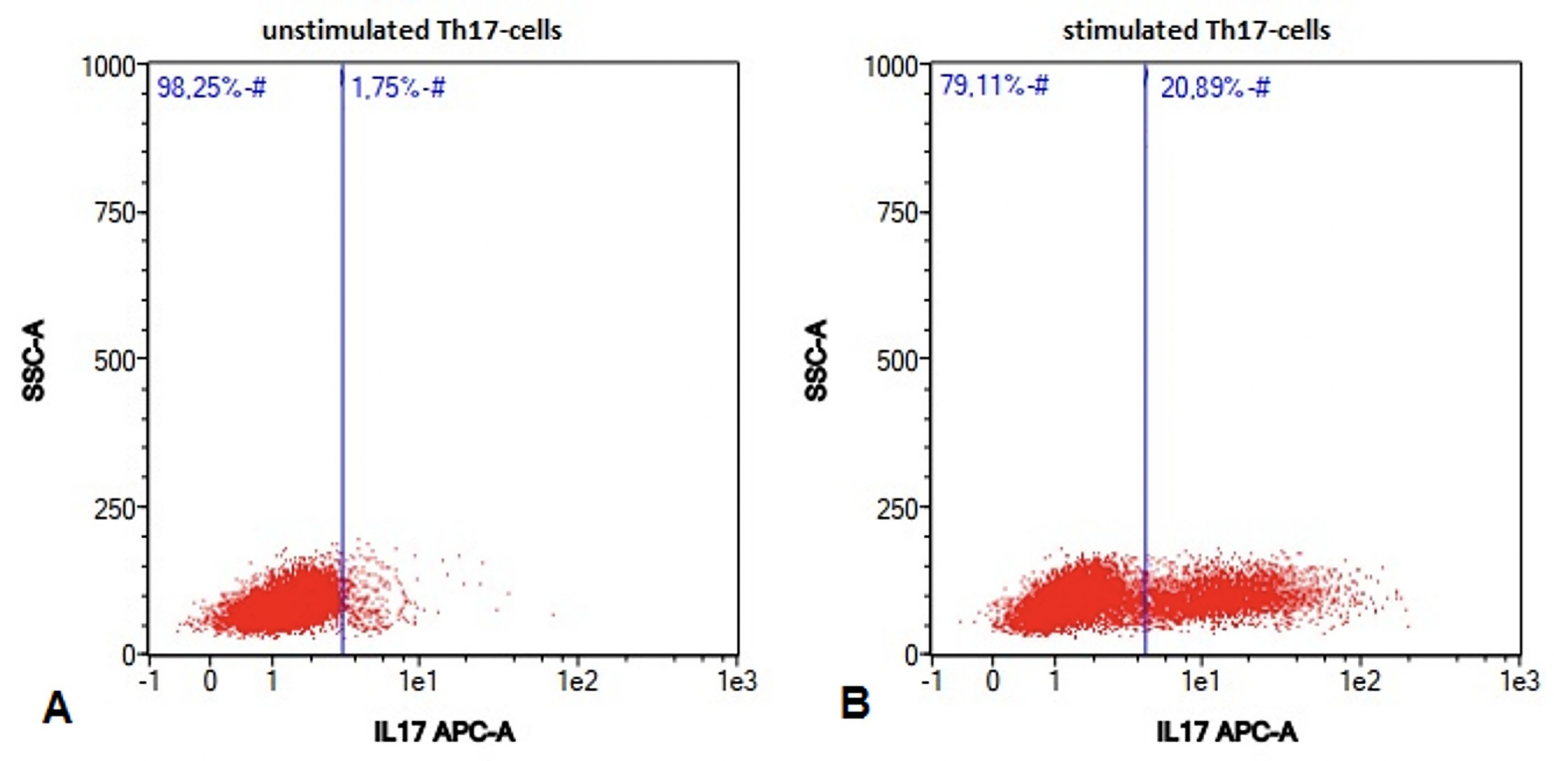
Stimulation of Th17-cells. Example of unstimulated Th17-cells (A) and stimulated Th17-cells (B) in canine peripheral blood sample of a dog suffering from intervertebral disc herniation measured by using flow cytometry. The stimulation of the Th17-cells was performed by using a specific lymphocyte medium including PMA (Phorbol-12-myristat-13-acetat) and Ionomycin calcium salt. Due to stimulation, it was possible to detect significant differences between the samples. IL17: Interleukin 17; Th17-cells: T-helper 17 cells; SSC: Sidewards Scatter; APC: Allophycocyanin.

**Fig 2:**
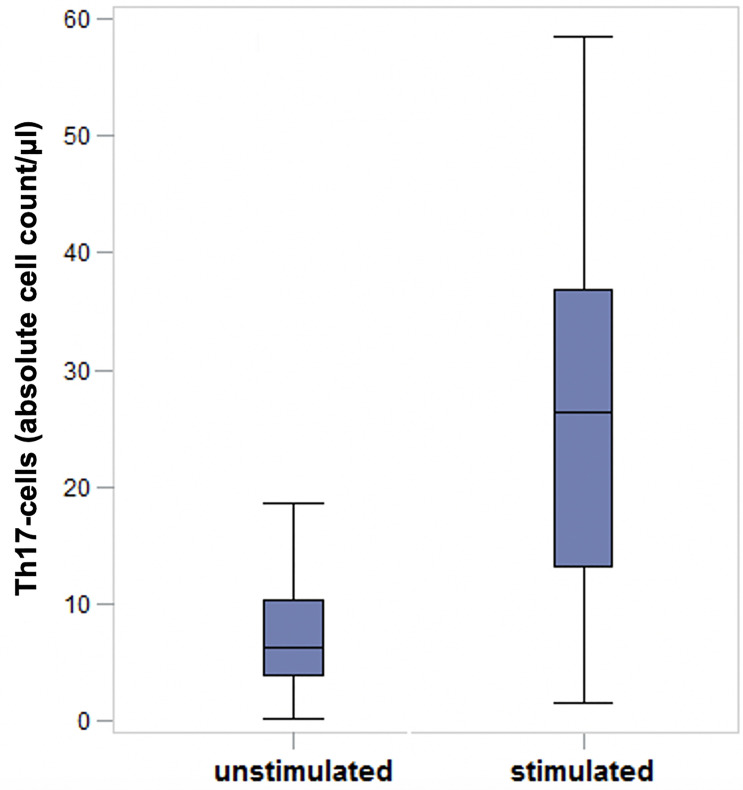
Results of unstimulated and stimulated Th17 cells of dogs suffering from IVDH (preoperatively). After cell stimulation with PMA, Ionomycin and Brefeldin A in peripheral blood samples of dogs suffering from intervertebral disc herniation (preoperatively) the number of Th17-cells was significantly higher in stimulated samples (p < 0.0001). Stimulation of Th-17 cells results in better analyzability and thus more exact measurements which makes it possible to detect significant differences. 62 samples consisting of each, a stimulated and an unstimulated sample = 124 samples in total.). Th17-cells: T-helper 17 cells; IVDH: Intervertebral disc herniation; μl: microliters.

The investigation of Th17-cells by using multicolor flow cytometry was feasible after lymphocyte isolation, stimulation of Th17-cells with PMA, Ionomycin and Brefeldin A, followed by staining the cells with antibodies. Highly significant differences could be determined between unstimulated and stimulated cells (Figs 1 and 2).

### Comparison of Th17-cells between IVDH group and healthy control group

**Fig 3:**
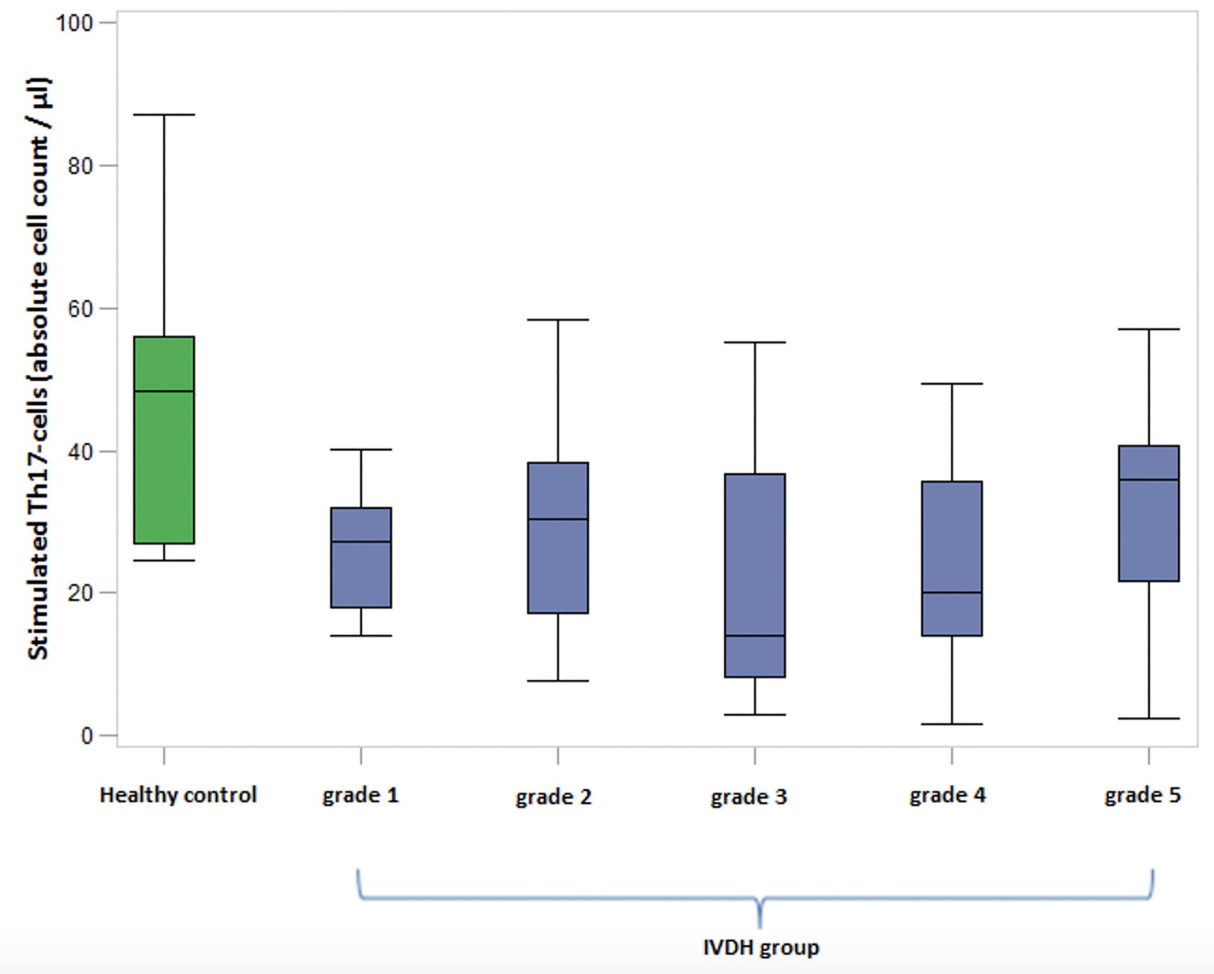
Th17-cells in comparison of severity grades of dogs suffering from IVDH and the healthy control group. Absolute number of stimulated Th17-cells/μl in canine blood with different severity of neurological signs. Green boxplot: Healthy control group; Blue boxplots: IVDH group measured preoperatively, classified by the grading system (grade 1–5) of Sharp and Wheeler (2005). The cell counts of the healthy dogs were significantly higher than those of the sick dogs (p < 0.001). No significant differences could be identified between the severity grades of the dogs affected by IVDH. The Th17-cell amount was generally reduced within the affected group in comparison with the healthy control group. Healthy control group: n = 10; IVDH group: 62 dogs (grade 1: n = 9; grade 2: n = 18; grade 3: n = 22; grade 4: n = 8; grade 5: n = 5). Th17-cells: T-helper 17 cells; IVDH: Intervertebral disc herniation; μl: microliters.

Highly significant differences between Th17-cell-levels could be determined between dogs suffering from IVDH and healthy control group: Preoperative Th17-cell values were significantly lower in dogs with IVDH than in healthy dogs (p < 0.001; Fig 3).

It is remarkable that Th17-cells levels in all groups are significantly lower than in the healthy dogs, regardless of the severity grade. Thus, prior to any surgery or conservative treatment Th17-cell levels are significantly lower than in healthy dogs (p < 0.001; Fig 3).

### Comparison of measurement time points

**Fig 4:**
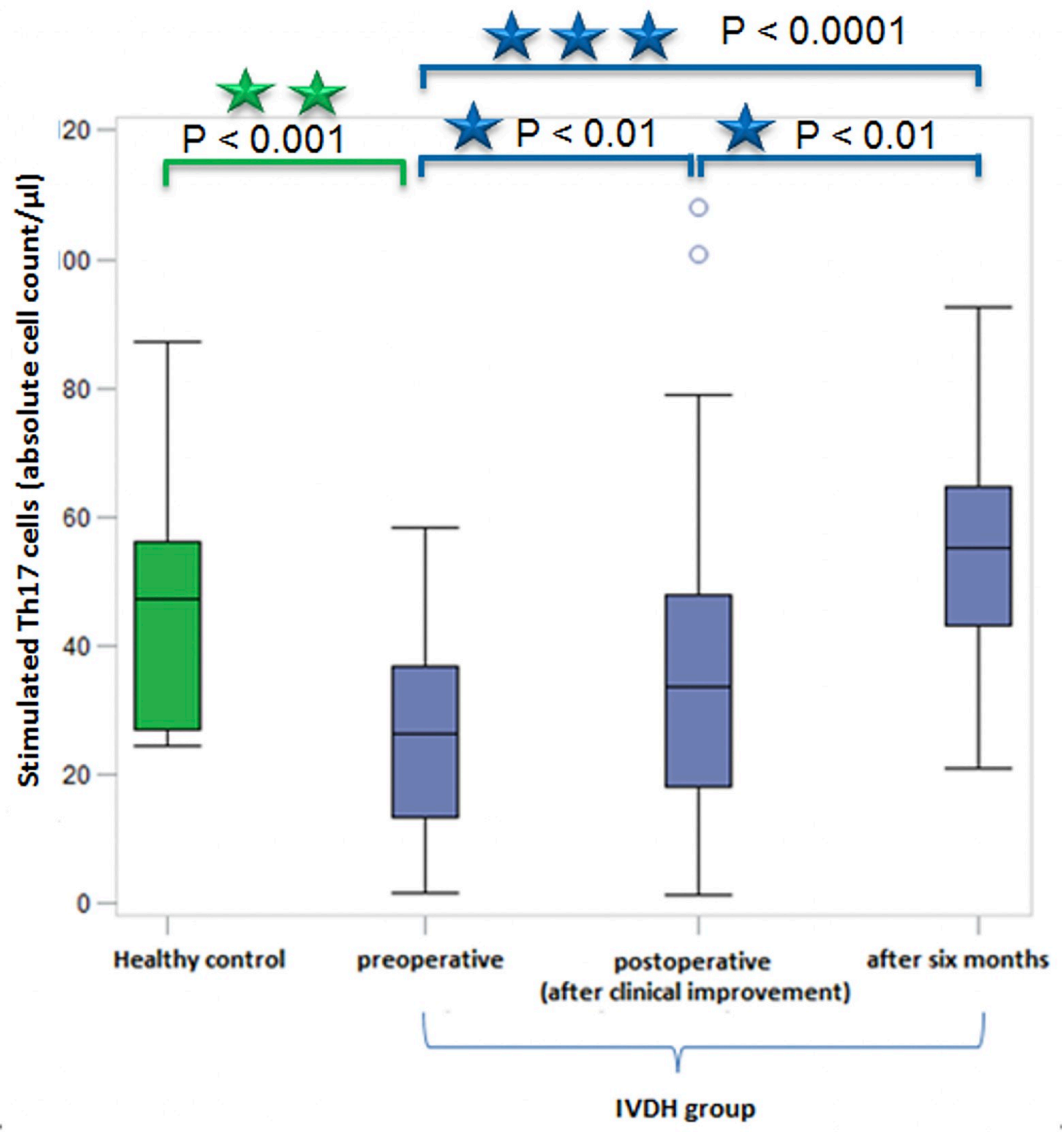
Significant differences between Th17-cell levels of dogs suffering from IVDH at all three sampling time points and in comparison with the healthy control group. Highly significant differences between preoperative Th17-cell levels and control after six month (***p < 0.0001). Significant differences between preoperative and postoperative Th17-cell levels and also between postoperative and after six month control Th17-cell levels (** p < 0.01). The difference between preoperative Th17-cell levels of dogs suffering from IVDH and healthy control group was high significant. ***: Highly significant (p < 0.0001); **: High significant (p < 0.001); *: Significant (p < 0.01). Green boxplot: Healthy control group; Blue boxplots: IVDH group sorted by measurement time; Sample number: Healthy control group: n = 10; IVDH group: n = 137 (preoperative: n = 62; postoperatively: n = 55; after six month: n = 20). Th17 cells: T-helper 17 cells; IVDH = intervertebral disc herniation; μl: microliters.

The following highly significant differences of stimulated Th17-cell levels in EDTA-blood samples could be determined between the three sampling time points: preoperatively, postoperatively after clinical improvement and after six months (Table 2 and Fig 4).

**Table 2:** Stimulated Th17-cells measured in EDTA-blood of dogs suffering from IVDH at three different sampling time points in comparison with Th17-cell levels of the healthy control group p-values of stimulated Th17-cells (absolute cell count/μl) in peripheral blood.

Effect	MTP	MTP	Pr >|t|	Adj P	Estimate	SE	DF	t-Value
MTP	Pre	H	0.0002					
	Post	H	0.1824					
	C	H	0.2240					
	Pre	C	< .0001	< .0001	26.9670	4.2942	73	6.28
	Pre	Post	0.0004	0.001	10.0810	2.9388	73	3.74
	Post	C	0.0005	0.0014	15.9860	4.3647	73	3.66

Preoperatively (Pre), postoperatively after clinical improvement (Post), control after six months (C), healthy control (H); measurement time point (MTP); adjust P-value (Adj P) for multiple comparisons (adjustment: Tukey-Kramer); Standard Error (SE); number of degrees of freedom (DF); Pr >|t| represents the two-tailed p-value computed by using the t distribution.

The Th17-cell values of dogs were lowest prior to any therapy. After recovery and clinical improvement new blood samples were obtained. We detected that the Th17-cell levels were significantly higher compared to the values measured prior to therapy (p < 0.01). After 6 months another significant increase of the Th17-cell levels was found (p < 0.01). The most significant difference could be found between Th17-cell levels of dogs with acute IVDH prior to therapy and levels of recovered dogs (p < 0.0001).

### Comparison IVDH group and healthy control group

In addition, we compared Th-17 values of dogs suffering from acute IVDH and recovered dogs with the data of a group of clinically healthy dogs.

Highly significant differences could be found between Th17-cell values of dogs suffering from IVDH (preoperatively) and the healthy control group (p < 0.001). During the recovery period, Th17-cell levels of the IVDH group converged towards the values of the healthy control group. No significant differences between IVDH group (postoperatively, control after six months) and healthy control group could be observed (Table 2).

Preoperatively, Th17-cell levels were strongly decreased in contrast to healthy controls. The decreased number of Th17-cells recovered postoperatively so that Th17-cell values were comparable to the healthy control group after six months (Table 2 and Fig 4).

#### Comparison of Th17-cell levels of acute and chronic intervertebral disc herniation

In the current study dogs suffering from acute (< 24 days, n = 49) as well as chronic IVDH (>24 days; n = 13) were included. However, no difference between chronic and acute disease and Th17-cell levels was detected (p > 0.1). Both groups were statistically significant different to healthy dogs.

**Comparison of Th17-cell values with respect to the course of the disease.** There were also no significant differences between Th17-cell levels regarding the outcome of the disease (improvement (n = 49), unchanged condition (n = 3), deterioation (n = 3); p > 0.1). Due to the small number of dogs within this study, whose health condition did not improve, critical reflection of the study’s results is advisable.

#### Results of IL-17 concentration measured in serum and CSF samples

In addition to Th17-cells measurements twenty-seven paired serum and CSF samples were collected (IVDH group n = 17; healthy group n = 10) for IL-17 assessment. All samples were analyzed by using Enzyme linked immunosorbent assays (ELISA) simultaneously.

**Fig 5:**
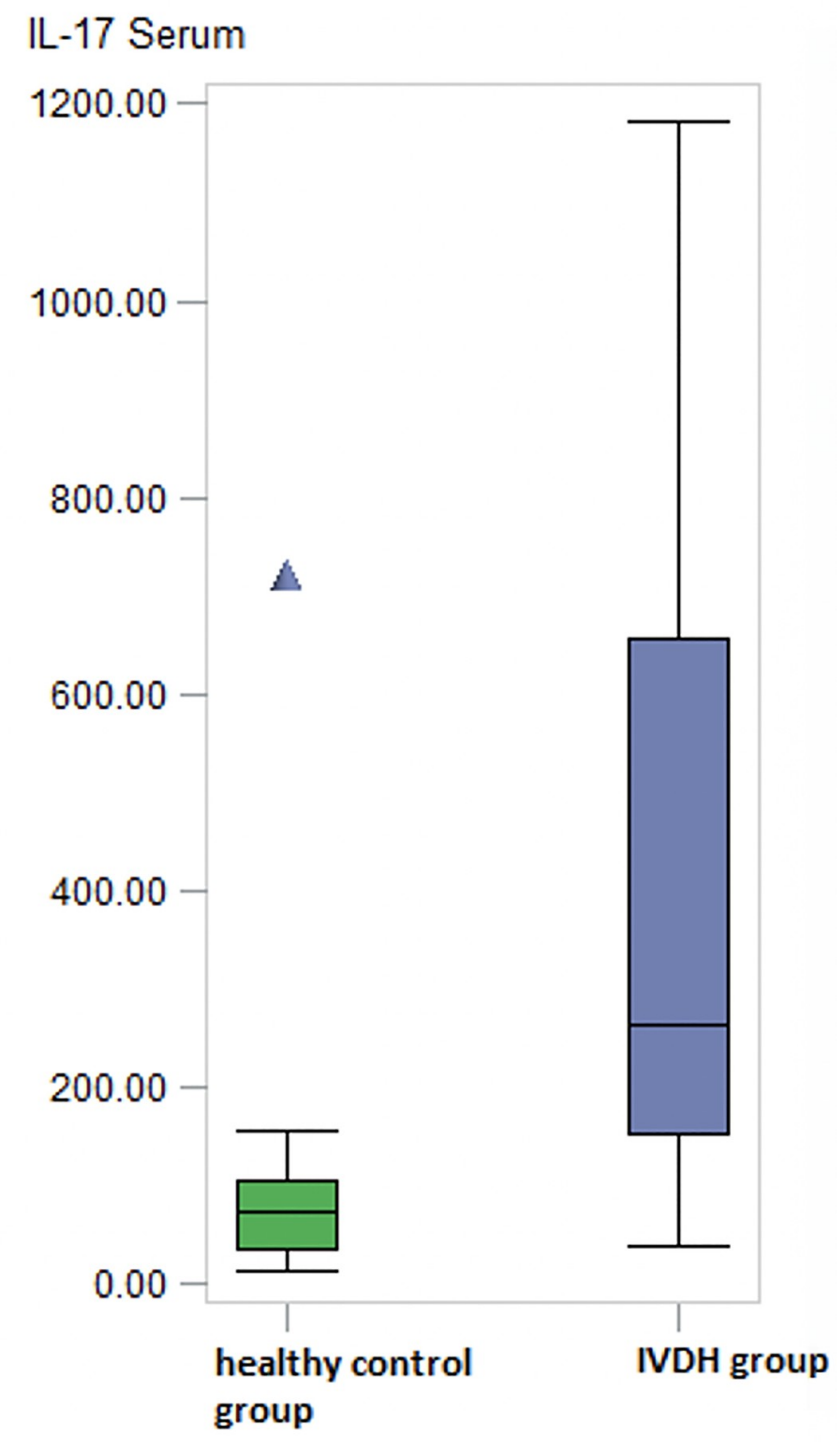
IL 17 levels in serum. Significantly higher levels of IL-17 pg/ml measured in serum preoperatively of dogs suffering from IVDH in comparison to healthy controls (p < 0.01). Sample number: Healthy control group: n = 10; IVDH group: n = 23. IVDH = intervertebral disc herniation; IL-17: Interleukin-17; pg/ml: picogram/milliter.

Compared to the healthy controls, IL-17 measured in serum (preoperatively) was significantly higher (p = 0.0025) in dogs with IVDH (Table 3 and Fig 5).

**Table 3:** IL-17 levels measured in serum (preoperatively).

Effect	N	Range (min - max) pg/ml	Median pg/ml
IVDH group	23	39.67–1182.78	262.32
Healthy group	10	14.99–722.27	74.26

IVDH: Intervertebral disc herniation; IL-17: Interleukin-17; pg/ml: picogram/milliter.

**Fig 6:**
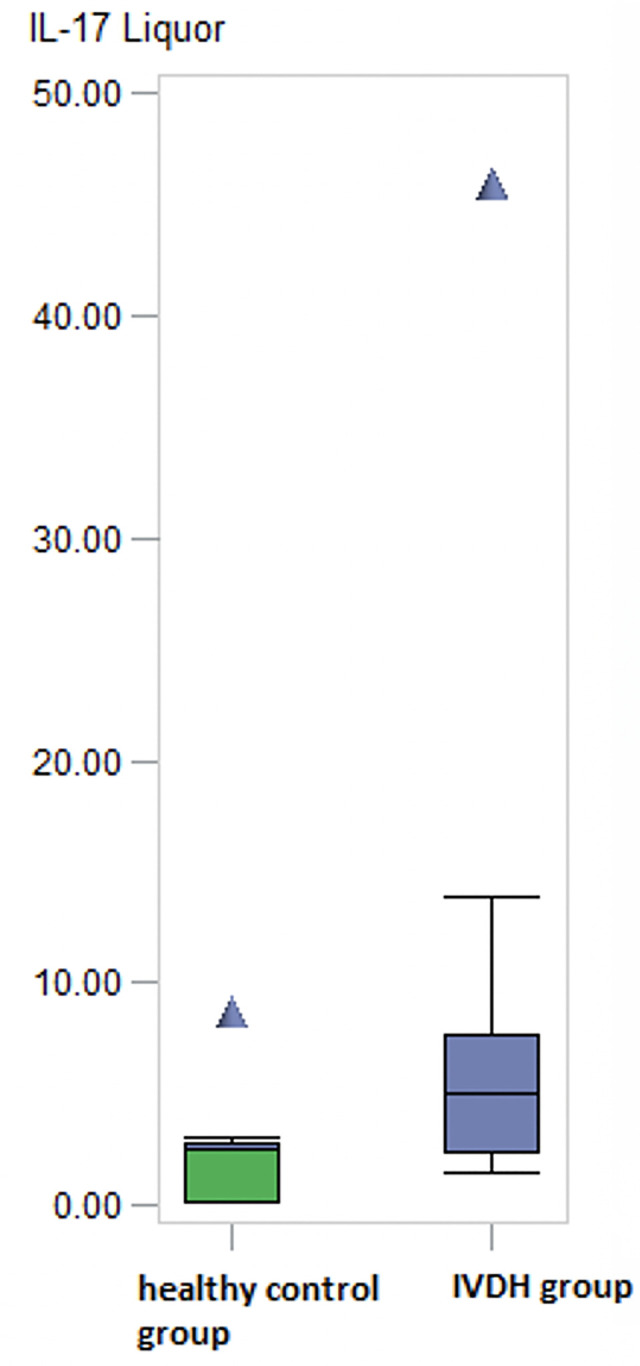
IL 17 levels in CSF. No considerable difference of IL-17 pg/ml levels in CSF between the groups (p > 0.05) Sample number: Healthy control group: n = 10; IVDH group: n = 17; IVDH: Intervertebral disc herniation; IL-17: Interleukin-17; CSF: Cerebrospinal fluid; pg/ml: picogram/milliter.

However, there was no considerable difference between IL-17 levels in CSF among the groups (p = 0.2047), even CSF levels in dogs with IVDH had slightly higher values (Table 4 and Fig 6).

**Table 4:** IL-17 levels measured in CSF (preoperatively).

Effect	N	Range (min—max) pg/ml	Median pg/ml
IVDH group	17	1.50 - 46.00	5.06
Healthy group	10	0.10 - 8.76	2.53

IVDH: Intervertebral disc herniation; IL-17: Interleukin-17; CSF: Cerebrospinal fluid; pg/ml: picogram/milliter, herniation; IL-17: Interleukin-17; CSF: Cerebrospinal fluid; pg/ml: picogram/milliter.

All results are summarized within the following table (Table 5) and raw data are supplied (S1 Table).

**Table 5:** Stimulated Th17-cell levels in EDTA-blood, IL-17 concentrations in serum and cerebrospinal fluid (CSF) in dogs suffering from intervertebral disc herniation (IVDH) and in healthy control dogs.

	Preoperatively	Postoperatively (after clinical improvement)	Follow up after six months	Healthy control group
**Th17-cells**	n = 62	n = 55	n = 20	n = 10
(absolute cell	median: 26.34	median: 33.79	median: 55.23	median: 48.39
count/μl)	(range: 1.59 – 58.48)	(range: 1.42 – 109.96)	(range: 20.96 – 92.7)	(range: 25.52 – 87.28)
	n = 23*	–	–	n = 10
**IL-17 serum**	median: 262.32			median: 74.26
levels	range: 102.25 – 1182.78			range: 14.99 – 155.06
(pg/ml)	(outlier: 39.67)			(outlier: 722.27)
	n = 17	–	–	n = 10
**IL-17 CSF**	median: 5.06			median: 2.53
levels	range: 1.5 – 13.87			range: 0.1 – 3.04
(pg/ml)	(outlier: 46)			(outlier: 8.76)

Data is provided as median and range. *paired serum and CSF samples: n = 17.

## Discussion

The primary objective of this study was to examine if Th17-cells are involved in the pathogenesis of IVDH in dogs. This hypothesis could be confirmed. Significantly decreased blood Th17-cell levels and increased serum IL-17 levels were determined simultaneously in dogs with acute IVDH in comparison to healthy dogs. These findings lead to the assumption that in an acute phase of IVDH a high activity and consequential consumption of IL-17-producing cells can be suspected.

Intervertebral disc herniation is a common naturally occurring disorder of the vertebral column primarily seen in chondrodystrophic dogs but can also affect any other dog breed [32,33]. The most common breeds in this study suffering from IVDH were french bulldog (n = 12; 19.4%) and dachshund (n = 12; 19.4%) to equal parts. These results are in concordance to findings of previous studies [1,13,34].

The prognosis of this disease depends on the severity of SCI, the degree of neurological deficits as well as correct and swift treatment [10,13,35]. However, a reliable prognosis can be challenging, especially concerning paraplegic dogs without deep pain perception [7]. IVDH in dogs is often accompanied with postoperative intensive care in of paraplegic dogs with a long time of rehabilitation [12]. Thus, there is a need to improve prognostic options. Currently, the prognosis of IVDH is mostly based on presence or absence of deep pain perception [6,7]. Objective, directly measurable parameters such as biomarkers are desirable for this disease to support decision making and discussing a reliable prognosis with dog owners.

Biomarkers are objective and reproducible measurable parameters of biological processes which represent a health condition without being influenced by subjective impressions [8]. The World Health Organization (WHO) defined a biomarker as: “[…] any substance, structure or process that can be measured in the body or its products and influence or predict the incidence of outcome or disease […]” [8]. In the last years biomarkers have greatly gained importance in clinical research and clinical practice. Search for prognostic biomarkers for dogs with IVDH is ongoing [36,37].

To examine if Th17-cells are involved in the pathogenesis of IVDH in dogs and furthermore if these cells might represent a potential biomarker for this disease an easily applicable, minimal-invasive, non-surgical procedure was preferred. For this purpose, we used a protocol by KOL et al. who demonstrated that Th17-cell levels can be measured in peripheral blood [27]. We modified this protocol in order to establish a more practicable approach for daily routine [28,29]. Blood was taken prior to decompressive surgery to measure Th17-cells and IL-17 levels avoiding any influence of iatrogenic interventions. Follow up samples were taken after the surgical approach when dogs improved clinically. A certain modification of the number of Th17-cells and IL-17 levels by the surgical approach alone cannot be excluded at this second time point of blood sampling. Therefore several dogs were examined in addition 6 months after surgery.

In 2005 IL-17-producing Th17-cells were described for the first time [38,39]. A proinflammatory effect of IL-17 and IL-17F is suspected in human psoriasis and rheumatic arthritis and both support the expression of proinflammatory mediators [40–42]. Furthermore, IL-17 is suspected of being involved in the pathogenesis of several autoimmune diseases [19]. Increased IL-17 levels were found in psoriasis, inflammatory bowel disease (IBD), rheumatoid arthritis, asthma and multiple sclerosis [18,19]. Decreased IL-17 levels can be observed in B-cell non-hodgkin’s lymphoma and cellular immune deficits [43]. Also, in veterinary medicine IL17 influencing processes are suspected in dog diseases. KOL et al. detected IL-17-producing cells in several inflamed tissues and blood, particularly with respect to chronic inflammatory diseases of dogs such as IBD, gingivitis, rhinitis and necrotizing meningoencephalitis [27]. The highest values could be measured in the intestinal mucosa of dogs with IBD and in the gingiva of dogs suffering from chronical gingivitis [27].

FREUNDT-REVILLA et al. found increased intrathecal levels of IL-17 in blood of dogs suffering from an acute phase of SRMA and after relapses [23]. Control groups also included dogs with IVDH which showed increased IL-17 levels in blood as well.

Understanding the possible influence of Th17-cells on the course of SCI would allow the development of novel therapeutic approaches based on specific cytokine modulation. To our knowledge no studies investigated IL-17-producing cells in blood and CSF concerning their influence on IVDH in dogs until now.

Human studies already confirmed an involvement of Th17-cells in human IVDH [17]. SHAMJI et al. were able to determine a high expression of lL-17 in degenerated and herniated disc material and suspected a Th17-mediated process in the pathogenesis of human IVDH [17]. In contrast, IL-17 in healthy disc material was almost absent. Those results support the hypothesis that there is an immune system activation at the time of disc herniation. SPITZBARTH et al. demonstrated that dogs represent an applicable model for human SCI, thus leading to the assumption that the pathogeneses of both species are of similar nature [24]. The authors could detect a proinflammatory environment [24].

The findings of SHAMJI et al. are in accordance with the results of this study [17]. We also determined significantly higher levels of IL-17 in serum of dogs during an acute phase of IVDH. In contrast to healthy controls IL-17 levels measured in serum were strongly increased preoperatively.

CHENG et al. concluded that human patients with ruptured annulus fibrosus showed a maximum of pain intensity along with significantly higher Th17-cell levels in contrast to patients with non-ruptured annulus fibrosus or in healthy controls, which showed the lowest values [25]. Accordingly, pain intensity was positively correlated with increased levels of Th17-cells and IL-17 expression. These research findings lead to the assumption that IL-17-producing T-helper cells trigger an inflammatory reaction and are responsible for the development and maintenance of pain [44,45].

In contrast to CHENG et al. we used the absolute cell numbers of Th17-cells instead of percentage values in order to include blood count changes such as lymphopenia or lymphocytosis [25]. Thus, the results of the current study are not entirely comparable to the results of other studies using percentage values.

All the affected dogs in this study showed signs of pain. Exact pain scoring was not feasible in the context of the current study. Thus, a correlation between Th17/IL-17 levels and higher pain scores could not be determined. For this purpose, a standardized pain scoring examination would have been necessary.

We did investigate, however, if there was a correlation between stimulated Th17-cell values and the severity grades defined by SHARP and WHEELER and we could not determine any correlation between Th17-cell numbers and the severity grades [3]. Both, dogs with ambulatory paresis as well as plegic dogs with or without deep pain perception showed significantly lower Th17-cell levels preoperatively in comparison to healthy controls. However, no differences between the severity grades and the respective Th17-cell values could be detected. A possible reason might be that any severity grade is correlated with an acute inflammatory reaction and Th17-cells are stimulated to produce IL-17 from the very beginning of a disease resulting in a slow recovery of the cells. The slightly increased Th17-cell numbers after recovery from the disease correlate with this assumption.

This study reveals new insights into the pathogenesis of IVDH in dogs. The decreased number of absolute Th17-cells may be caused by high activity and consequential cell consumption as a result of increased IL-17 production. Our assumption is that there is a high activity of Th17-cells producing IL-17 in an acute phase of IVDH. Thus, Th17-cells are consumed in this process or were exhausted after IL-17 producing so that they did not survive the dyeing process or rather were not able to get stimulated for producing IL-17.

For further identification of the influence of Th17-cells in the pathogenesis of IVDH, additional investigations are necessary. For this purpose, the next step would be to analyze the course of IL-17 in serum to verify if there is a correlation between decreased Th-17 cell numbers and increased IL-17 at different measurement time points. This might lead to the assumption that an acute phase of IVDH is accompanied with an elevated usage or consumption of Th17-cells due to increased IL-17 production.

In addition, it would be interesting to examine the ratio between Th17-cells and regulatory T-cells (T_reg_) to see if there is an irregular Th17/T_reg_ balance as it can be observed in humans with chronic low back pain [44].

LUCHTING et al. discovered an altered T_reg_/Th17 ratio in patients with chronic low back pain [44]. Anti-inflammatory T_reg_-cells were increased while Th17-cell levels decreased as in the current canine study. The assumption of LUCHTING et al. was, that either the duration of pain and stress might induce a dysregulation of the Th17/T_reg_ immune cell balance or, on the contrary, that the dysregulation occurs first. A dysregulation might result in a clinical chronification of pain symptoms which is supported by their additional findings that the imbalance persisted even after six months follow-up [44].

The first assumption, that the dysregulation might be induced by a prolonged period of the disease causing decreased Th17-cell numbers would correlate with the findings of our study and support the hypothesis of a stress induced immune cell response and subsequent consumption of Th17-cells in dogs with IVDH.

In contrast to LUCHTING et al., in dogs Th17-cell numbers recovered after six months follow-up which contradicts the theory of the occurrence of an immune cell disorder prior to clinical pain symptoms [44]. However, it should be noted, that IVDH in human patients is often not managed surgically but treated by application of analgesics [46,47]. In such an approach the herniated disc material is not removed and could lead to continuously decreased Th17-cell numbers.

In summary, Th17-cells could play an important role in the pathogenesis of SCI due to IVDH and the subsequent clinical signs. In addition, Th17-cells might influence the endogenous recovery from neuropathic pain. However, further investigations are required to develop better treatment options [48]. In human medicine, there are promising results with anti-IL-17 monoclonal antibodies in psoriasis treatment [49]. At presence of a proven Th17-induced pain reaction concerning IVDH, these monoclonal antibodies could also offer a new therapeutic approach.

## Conclusion

The purpose of the current study was to determine a possible role of Th17-cells in the pathogenesis of IVDH.

The most obvious finding emerging from this study is that Th17-cell levels were significantly decreased in an acute phase of IVDH and recovered postoperatively which supports the hypothesis that Th17-cells are involved in the pathogenesis of IVDH and is consistent with a high consumption of Th17-cells. Likewise, the high IL-17 level in serum samples indicates a high activity of Th17-cells in dogs suffering from IVDH.

Accordingly, a high activity/usage or consumption of IL-17-producing Th17-cells is suspected in acute IVDH. However, a correlation of Th17-cells with the outcome of the disease regarding the prognosis could not be confirmed.

These findings offer additional information regarding the pathogenesis of canine IVDH and open the discussion about additional new treatment strategies.

## Supporting information

S1 Table S1 Tables 1 and 2. **Study data of the dogs suffering from IVDH (Intervertebral Disc Herniation) and Study data of the healthy control group.** PN: patient number; BA: Beagle; DA: Dachshund; DAL: Dalmatian; FB: French Bulldog; HA: Havanese; HB: Hanoverian Bloodhound; JR: Jack Russell Terrier; SHD: Short-haired Dachshund; LHD: Long-haired Dachshund; LR: Labrador Retriever; MA: Maltese; MB: Mixed breed dog; PU: Pug; PO: Poodle; RHD: Rough-haired Dachshund; RO: Rottweiler; ST: Shi Tzu; SP: Spaniel; WCP: Welsh Corgie Pembroke; YT: Yorkshire Terrier; MS: Miniature Schnauzer; M: male; Mn: male neutered; F: female, Fn: female neutered; kg: kilogram; PRE Th17 S: stimulated Th17-cells (absolute cell count/ μl) before start of treatment (preoperative); PRE Th17 UN: unstimulated Th17-cells (absolute cell count/ μl) before start of treatment (preoperative); POST Th17 S: stimulated Th17-cells (absolute cell count/ μl) postoperative after clinical improvement; POST Th17 UN: unstimulated Th17-cells (absolute cell count/ μl) postoperative after clinical improvement; CO Th17 S: stimulated Th17-cells (absolute cell count/μl) at control examination after 6 months; CO Th17 UN: unstimulated Th17-cells (absolute cell count/μl) at control examination after 6 months; IL-17: Interleukin-17; μl: microliter; pg/ml: picogram/milliter; IVDH: Intervertebral Disc Herniation. PN: patient number; BA: Beagle; M: male; F: female; kg: kilogram; Th17 S: stimulated Th17-cells (absolute cell count/ μl); Th17 UN: unstimulated Th17-cells (absolute cell count/ μl); IL-17: Interleukin-17; μl: microliter; pg/ml: picogram/milliter.
(DOCX)
