# Supplementary material for: Evaluation of the involvement of Th17-cells in the pathogenesis of canine spinal cord injury
Source: PLoS One. 2021 Sep 30;16(9):e0257442. doi: 10.1371/journal.pone.0257442 (PMC8483396; doi:10.1371/journal.pone.0257442)
Supplement: S1 Table — Study data of the dogs suffering from IVDH (Intervertebral Disc Herniation) and Study data of the healthy control group. PN: patient number; BA: Beagle; DA: Dachshund; DAL: Dalmatian; FB: French Bulldog; HA: Havanese; HB: Hanoverian Bloodhound; JR: Jack Russell Terrier; SHD: Short-haired Dachshund; LHD: Long-haired Dachshund; LR: Labrador Retriever; MA: Maltese; MB: Mixed breed dog; PU: Pug; PO: Poodle; RHD: Rough-haired Dachshund; RO: Rottweiler; ST: Shi Tzu; SP: Spaniel; WCP: Welsh Corgie Pembroke; YT: Yorkshire Terrier; MS: Miniature Schnauzer; M: male; Mn: male neutered; F: female, Fn: female neutered; kg: kilogram; PRE Th17 S: stimulated Th17-cells (absolute cell count/ μl) before start of treatment (preoperative); PRE Th17 UN: unstimulated Th17-cells (absolute cell count/ μl) before start of treatment (preoperative); POST Th17 S: stimulated Th17-cells (absolute cell count/ μl) postoperative after clinical improvement; POST Th17 UN: unstimulated Th17-cells (absolute cell count/ μl) postoperative after clinical improvement; CO Th17 S: stimulated Th17-cells (absolute cell count/μl) at control examination after 6 months; CO Th17 UN: unstimulated Th17-cells (absolute cell count/μl) at control examination after 6 months; IL-17: Interleukin-17; μl: microliter; pg/ml: picogram/milliter; IVDH: Intervertebral Disc Herniation. PN: patient number; BA: Beagle; M: male; F: female; kg: kilogram; Th17 S: stimulated Th17-cells (absolute cell count/ μl); Th17 UN: unstimulated Th17-cells (absolute cell count/ μl); IL-17: Interleukin-17; μl: microliter; pg/ml: picogram/milliter. (DOCX) [file pone.0257442.s001.docx]

**S1 Table 1 and 2. Study data of the dogs suffering from IVDH (Intervertebral Disc Herniation) and Study data of the healthy control group**

| \| **PN** \| **Breed** \| **Age**  (in years) \| **Sex** \| **Weight** (kg) \| **Severity grade** \| **PRE Th17 S**  (absolute cell count/μl) \| **PRE Th17 UN**  (absolute cell count/μl) \| **POST Th17 S**  (absolute cell count/μl) \| **POST Th17 UN**  (absolute cell count/μl) \| **CO**  **Th17 S**  (absolute cell count/μl) \| **CO**  **Th17 UN** (absolute cell count/μl) \| **IL-17 Serum** (pg/ml) \| **IL-17 Liquor** (pg/ml) \| \| --- \| --- \| --- \| --- \| --- \| --- \| --- \| --- \| --- \| --- \| --- \| --- \| --- \| --- \| \| **1** \| DA \| 6 \| Fn \| 2.6 \| 3 \| 9.21 \| 0.8 \| 16.1 \| 3.85 \| - \| - \| - \| - \| \| **2** \| MB \| 5 \| Mn \| 4.4 \| 2 \| 14.72 \| 1.62 \| 29.98 \| 10.99 \| 64.64 \| 10.25 \| - \| - \| \| **3** \| DA \| 11 \| M \| 4.5 \| 3 \| 6.05 \| 3.12 \| 26.85 \| 2.84 \| - \| - \| - \| - \| \| **4** \| FB \| 2 \| M \| 4.6 \| 3 \| 10.95 \| 9.27 \| 1.42 \| 0.55 \| - \| - \| - \| - \| \| **5** \| WCP \| 12 \| Mn \| 5.0 \| 3 \| 33.04 \| 6.89 \| 49.49 \| 7.49 \| - \| - \| 262.32 \| 6.07 \| \| **6** \| DA \| 4 \| M \| 5.1 \| 3 \| 3.7 \| 1.63 \| 10.12 \| 16.24 \| - \| - \| - \| - \| \| **7** \| FB \| 4 \| F \| 5.2 \| 2 \| 49.95 \| 10.61 \| 17.12 \| 3.59 \| 69.42 \| 17.46 \| 157.87 \| 9.05 \| \| **8** \| FB \| 3 \| Fn \| 5.6 \| 2 \| 38.59 \| 7.88 \| - \| - \| 44.31 \| 6.65 \| - \| - \| \| **9** \| DA \| 8 \| Fn \| 5.8 \| 3 \| 14.85 \| 5.74 \| - \| - \| - \| - \| - \| - \| \| **10** \| MB \| 7 \| Mn \| 6.0 \| 3 \| 3.1 \| 0.82 \| 12.74 \| 4.49 \| 52.5 \| 3.84 \| - \| - \| \| **11** \| MB \| 8 \| Mn \| 6.2 \| 2 \| 30.97 \| 4.38 \| 21.01 \| 4.44 \| 64.57 \| 8.46 \| - \| - \| \| **12** \| JR \| 12 \| Mn \| 6.5 \| 3 \| 12.75 \| 1.58 \| 25.99 \| 3.8 \| 20.96 \| 4.97 \| - \| - \| \| **13** \| BA \| 10 \| F \| 6.5 \| 2 \| 17.19 \| 4.62 \| 17.5 \| 5.09 \| - \| - \| - \| - \| \| **14** \| BA \| 9 \| Mn \| 6.9 \| 2 \| 26.39 \| 8.88 \| 38.22 \| 11.85 \| - \| - \| - \| - \| \| **15** \| ST \| 7 \| Mn \| 7.0 \| 3 \| 7.19 \| 1.2 \| 5.15 \| 2.22 \| - \| - \| - \| - \| \| **16** \| ST \| 5 \| M \| 7.4 \| 5 \| 21.69 \| 4.48 \| - \| - \| - \| - \| - \| - \| \| **17** \| LHD \| 7 \| Mn \| 7.9 \| 4 \| 15.19 \| 5.63 \| 14.23 \| 5.97 \| - \| - \| 238.2 \| 4.23 \| \| **18** \| DAL \| 6 \| M \| 7.9 \| 4 \| 49.3 \| 5.52 \| 26.42 \| 7.49 \| - \| - \| 618.26 \| 7.72 \| \| **19** \| JR \| 13 \| Mn \| 7.9 \| 2 \| 19.36 \| 3.89 \| - \| - \| - \| - \| - \| - \| \| **20** \| DA \| 11 \| Mn \| 8.3 \| 3 \| 8.31 \| 2.45 \| 63.51 \| 11.4 \| - \| - \| - \| - \| \| **21** \| MB \| 7 \| Fn \| 8.5 \| 3 \| 54.81 \| 9.32 \| 58.43 \| 20.95 \| 46.77 \| 8.78 \| - \| - \| \| **22** \| FB \| 9 \| Fn \| 9.2 \| 2 \| 7.84 \| 4.81 \| - \| - \| - \| - \| 294.4 \| - \| \| **23** \| LHD \| 3 \| F \| 9.2 \| 4 \| 34.95 \| 11.16 \| 39.93 \| 9.25 \| - \| - \| - \| - \| \| **24** \| PU \| 3 \| M \| 9.4 \| 5 \| 2.5 \| 0.59 \| 2.51 \| 0.4 \| 60.57 \| 8.85 \| - \| - \| \| **25** \| PO \| 10 \| Fn \| 9.4 \| 4 \| 1.59 \| 0.32 \| 36.34 \| 5.97 \| - \| - \| - \| - \| \| **26** \| LR \| 8 \| F \| 9.6 \| 2 \| 38.34 \| 7.79 \| 42.17 \| 11 \| 64.48 \| 8.16 \| 396.82 \| 46 \| \| **27** \| BA \| 9 \| Mn \| 9.6 \| 1 \| 40.22 \| 7.41 \| 29.7 \| 7.47 \| - \| - \| - \| - \| \| **28** \| JR \| 3 \| Fn \| 9.8 \| 2 \| 11.64 \| 5.5 \| 14.1 \| 4.13 \| - \| - \| - \| - \| \| **29** \| JR \| 6 \| M \| 10.9 \| 3 \| 42.36 \| 3.45 \| 38.43 \| 5.9 \| 41.91 \| 4.86 \| 163.55 \| - \| \| **30** \| FB \| 5 \| Fn \| 10.9 \| 3 \| 13.23 \| 4.06 \| 28.41 \| 4.38 \| - \| - \| - \| - \| \| **31** \| MB \| 11 \| Mn \| 10.9 \| 3 \| 10.23 \| 1.2 \| 47.95 \| 7.59 \| 41.93 \| 7.54 \| 773.87 \| - \| \| **32** \| FB \| 2 \| M \| 11.0 \| 5 \| 57.2 \| 9.75 \| 40.32 \| 11.99 \| - \| - \| 277.46 \| - \| \| **33** \| PO \| 11 \| M \| 11.2 \| 2 \| 40.64 \| 11.21 \| 78.93 \| 32.58 \| 47.26 \| 4.31 \| 143.58 \| 5.34 \| \| **34** \| YT \| 3 \| M \| 11.3 \| 2 \| 58.48 \| 12.12 \| 78.6 \| 26.25 \| 47.82 \| 22.04 \| - \| - \| \| **35** \| MB \| 6 \| Mn \| 11.3 \| 3 \| 36.65 \| 6.41 \| 62.97 \| 28.07 \| - \| - \| - \| - \| \| **36** \| LHD \| 2 \| F \| 11.4 \| 4 \| 22.8 \| 7.95 \| 28.34 \| 18.4 \| 61.85 \| 15.98 \| - \| - \| \| **37** \| MA \| 2 \| Fn \| 11,4 \| 3 \| 28.52 \| 11.66 \| 42.07 \| 10.79 \| 92.7 \| 8.1 \| - \| - \| \| **38** \| MB \| 6 \| M \| 11.7 \| 3 \| 21.93 \| 4.96 \| 107.96 \| 21.9 \| - \| - \| - \| - \| \| **39** \| DA \| 7 \| Mn \| 12.9 \| 3 \| 17.18 \| 4.19 \| 12.21 \| 7.34 \| - \| - \| 39.67 \| 2.4 \| \| **40** \| YT \| 9 \| Fn \| 13.4 \| 2 \| 35.95 \| 16.24 \| 15.96 \| 2.46 \| 24.17 \| 4.85 \| 913.7 \| 3 \| \| **41** \| HA \| 8 \| Mn \| 13.5 \| 3 \| 39.05 \| 11.29 \| 46.52 \| 18.23 \| - \| - \| - \| - \| \| **42** \| FB \| 3 \| Mn \| 13.6 \| 3 \| 8.33 \| 1.78 \| 37.76 \| 15.09 \| - \| - \| - \| - \| \| **43** \| MS \| 13 \| M \| 13.8 \| 1 \| 17.94 \| 11.44 \| 17.55 \| 7.31 \| - \| - \| 102.25 \| 1.5 \| \| **44** \| LR \| 10 \| M \| 14.0 \| 3 \| 55.21 \| 6.78 \| 100.77 \| 29.39 \| 82.23 \| 11.9 \| 657.34 \| - \| \| **45** \| RO \| 9 \| M \| 15.1 \| 1 \| 17.35 \| 3.66 \| 26.37 \| 11.93 \| - \| - \| 1182.78 \| 4.42 \| \| **46** \| RHD \| 4 \| Mn \| 15.1 \| 2 \| 13.83 \| 13.75 \| 40.07 \| 3.81 \| 39.16 \| 8.21 \| - \| - \| \| **47** \| MB \| 7 \| Mn \| 15.2 \| 5 \| 40.68 \| 7.4 \| 27.3 \| 5.35 \| 57.96 \| 6.79 \| - \| - \| \| **48** \| JR \| 4 \| M \| 15.5 \| 2 \| 29.4 \| 9.89 \| 26.12 \| 10.87 \| - \| - \| 308.29 \| - \| \| **49** \| SHD \| 4 \| F \| 15.8 \| 5 \| 35.96 \| 10.64 \| - \| - \| - \| - \| - \| - \| \| **50** \| DAL \| 11 \| M \| 17.4 \| 4 \| 36.52 \| 6.37 \| 33.79 \| 9.38 \| - \| - \| - \| - \| \| **51** \| FB \| 2 \| Fn \| 18.5 \| 4 \| 17.25 \| 4.69 \| 19.72 \| 6.96 \| - \| - \| 156.71 \| 8.13 \| \| **52** \| JR \| 9 \| F \| 19.3 \| 1 \| 14.02 \| 5.42 \| 46.72 \| 28.3 \| - \| - \| - \| - \| \| **53** \| SP \| 8 \| Fn \| 20.0 \| 1 \| 29.45 \| 16.84 \| - \| - \| 70.56 \| 19.87 \| 152.84 \| 1.5 \| \| **54** \| FB \| 5 \| Fn \| 20.7 \| 4 \| 12.79 \| 8.36 \| 18.11 \| 9.48 \| - \| - \| - \| - \| \| **55** \| DA \| 13 \| Mn \| 21.5 \| 3 \| 48.78 \| 17.73 \| 54.45 \| 19.07 \| - \| - \| - \| - \| \| **56** \| FB \| 5 \| F \| 24.1 \| 1 \| 38.05 \| 3.09 \| 60.54 \| 14.34 \| - \| - \| 1001 \| 13.87 \| \| **57** \| BA \| 9 \| Mn \| 25.0 \| 1 \| 27.33 \| 12.27 \| 40.31 \| 29.38 \| - \| - \| 241.65 \| 5.13 \| \| **58** \| HA \| 10 \| Fn \| 27.3 \| 2 \| 36.87 \| 18.66 \| 75.79 \| 32.09 \| - \| - \| - \| - \| \| **59** \| FB \| 3 \| M \| 31.9 \| 2 \| 30.09 \| 5.72 \| 56.74 \| 39.61 \| - \| - \| - \| - \| \| **60** \| FB \| 5 \| M \| 33.0 \| 1 \| 32.02 \| 9.25 \| 40.54 \| 5.88 \| - \| - \| 115.01 \| 2.07 \| \| **61** \| MB \| 4 \| M \| 40.0 \| 1 \| 26.3 \| 13.96 \| 60.25 \| 19.6 \| - \| - \| 787.56 \| 2.05 \| \| **62** \| HB \| 2 \| M \| 51.8 \| 2 \| 33.43 \| 10.42 \| 25.29 \| 8.09 \| - \| - \| 121.54 \| 5.06 \| |
| --- | --- | --- | --- | --- | --- | --- | --- | --- | --- | --- | --- | --- | --- | --- | --- | --- | --- | --- | --- | --- | --- | --- | --- | --- | --- | --- | --- | --- | --- | --- | --- | --- | --- | --- | --- | --- | --- | --- | --- | --- | --- | --- | --- | --- | --- | --- | --- | --- | --- | --- | --- | --- | --- | --- | --- | --- | --- | --- | --- | --- | --- | --- | --- | --- | --- | --- | --- | --- | --- | --- | --- | --- | --- | --- | --- | --- | --- | --- | --- | --- | --- | --- | --- | --- | --- | --- | --- | --- | --- | --- | --- | --- | --- | --- | --- | --- | --- | --- | --- | --- | --- | --- | --- | --- | --- | --- | --- | --- | --- | --- | --- | --- | --- | --- | --- | --- | --- | --- | --- | --- | --- | --- | --- | --- | --- | --- | --- | --- | --- | --- | --- | --- | --- | --- | --- | --- | --- | --- | --- | --- | --- | --- | --- | --- | --- | --- | --- | --- | --- | --- | --- | --- | --- | --- | --- | --- | --- | --- | --- | --- | --- | --- | --- | --- | --- | --- | --- | --- | --- | --- | --- | --- | --- | --- | --- | --- | --- | --- | --- | --- | --- | --- | --- | --- | --- | --- | --- | --- | --- | --- | --- | --- | --- | --- | --- | --- | --- | --- | --- | --- | --- | --- | --- | --- | --- | --- | --- | --- | --- | --- | --- | --- | --- | --- | --- | --- | --- | --- | --- | --- | --- | --- | --- | --- | --- | --- | --- | --- | --- | --- | --- | --- | --- | --- | --- | --- | --- | --- | --- | --- | --- | --- | --- | --- | --- | --- | --- | --- | --- | --- | --- | --- | --- | --- | --- | --- | --- | --- | --- | --- | --- | --- | --- | --- | --- | --- | --- | --- | --- | --- | --- | --- | --- | --- | --- | --- | --- | --- | --- | --- | --- | --- | --- | --- | --- | --- | --- | --- | --- | --- | --- | --- | --- | --- | --- | --- | --- | --- | --- | --- | --- | --- | --- | --- | --- | --- | --- | --- | --- | --- | --- | --- | --- | --- | --- | --- | --- | --- | --- | --- | --- | --- | --- | --- | --- | --- | --- | --- | --- | --- | --- | --- | --- | --- | --- | --- | --- | --- | --- | --- | --- | --- | --- | --- | --- | --- | --- | --- | --- | --- | --- | --- | --- | --- | --- | --- | --- | --- | --- | --- | --- | --- | --- | --- | --- | --- | --- | --- | --- | --- | --- | --- | --- | --- | --- | --- | --- | --- | --- | --- | --- | --- | --- | --- | --- | --- | --- | --- | --- | --- | --- | --- | --- | --- | --- | --- | --- | --- | --- | --- | --- | --- | --- | --- | --- | --- | --- | --- | --- | --- | --- | --- | --- | --- | --- | --- | --- | --- | --- | --- | --- | --- | --- | --- | --- | --- | --- | --- | --- | --- | --- | --- | --- | --- | --- | --- | --- | --- | --- | --- | --- | --- | --- | --- | --- | --- | --- | --- | --- | --- | --- | --- | --- | --- | --- | --- | --- | --- | --- | --- | --- | --- | --- | --- | --- | --- | --- | --- | --- | --- | --- | --- | --- | --- | --- | --- | --- | --- | --- | --- | --- | --- | --- | --- | --- | --- | --- | --- | --- | --- | --- | --- | --- | --- | --- | --- | --- | --- | --- | --- | --- | --- | --- | --- | --- | --- | --- | --- | --- | --- | --- | --- | --- | --- | --- | --- | --- | --- | --- | --- | --- | --- | --- | --- | --- | --- | --- | --- | --- | --- | --- | --- | --- | --- | --- | --- | --- | --- | --- | --- | --- | --- | --- | --- | --- | --- | --- | --- | --- | --- | --- | --- | --- | --- | --- | --- | --- | --- | --- | --- | --- | --- | --- | --- | --- | --- | --- | --- | --- | --- | --- | --- | --- | --- | --- | --- | --- | --- | --- | --- | --- | --- | --- | --- | --- | --- | --- | --- | --- | --- | --- | --- | --- | --- | --- | --- | --- | --- | --- | --- | --- | --- | --- | --- | --- | --- | --- | --- | --- | --- | --- | --- | --- | --- | --- | --- | --- | --- | --- | --- | --- | --- | --- | --- | --- | --- | --- | --- | --- | --- | --- | --- | --- | --- | --- | --- | --- | --- | --- | --- | --- | --- | --- | --- | --- | --- | --- | --- | --- | --- | --- | --- | --- | --- | --- | --- | --- | --- | --- | --- | --- | --- | --- | --- | --- | --- | --- | --- | --- | --- | --- | --- | --- | --- | --- | --- | --- | --- | --- | --- | --- | --- | --- | --- | --- | --- | --- | --- | --- | --- | --- | --- | --- | --- | --- | --- | --- | --- | --- | --- | --- | --- | --- | --- | --- | --- | --- | --- | --- | --- | --- | --- | --- | --- | --- | --- | --- | --- | --- | --- | --- | --- | --- | --- | --- | --- | --- | --- | --- | --- | --- | --- | --- | --- | --- | --- | --- | --- | --- | --- | --- | --- | --- | --- | --- | --- | --- | --- | --- | --- | --- | --- | --- | --- | --- | --- | --- | --- | --- | --- | --- | --- | --- | --- | --- | --- | --- | --- | --- | --- | --- | --- | --- | --- | --- | --- | --- | --- | --- | --- | --- | --- | --- | --- | --- | --- | --- | --- | --- | --- | --- | --- | --- | --- | --- | --- | --- | --- | --- | --- | --- | --- | --- | --- | --- | --- | --- | --- | --- | --- | --- | --- | --- | --- | --- | --- | --- | --- | --- | --- | --- | --- | --- | --- | --- | --- | --- | --- | --- | --- | --- | --- | --- | --- | --- | --- | --- | --- | --- | --- | --- | --- | --- | --- | --- | --- | --- | --- | --- | --- | --- | --- | --- | --- | --- | --- | --- | --- | --- | --- | --- | --- | --- | --- | --- | --- | --- | --- | --- | --- | --- | --- | --- | --- | --- | --- | --- | --- | --- | --- | --- | --- |

PN: patient number; BA: Beagle; DA: Dachshund; DAL: Dalmatian; FB: French Bulldog; HA: Havanese; HB: Hanoverian Bloodhound; JR: Jack Russell Terrier; SHD: Short-haired Dachshund; LHD: Long-haired Dachshund; LR: Labrador Retriever; MA: Maltese; MB: Mixed breed dog; PU: Pug; PO: Poodle; RHD: Rough-haired Dachshund; RO: Rottweiler; ST: Shi Tzu; SP: Spaniel; WCP: Welsh Corgie Pembroke; YT: Yorkshire Terrier; MS: Miniature Schnauzer; M: male; Mn: male neutered; F: female, Fn: female neutered; kg: kilogram; PRE Th17 S: stimulated Th17-cells (absolute cell count/ μl) before start of treatment (preoperative); PRE Th17 UN: unstimulated Th17-cells (absolute cell count/ μl) before start of treatment (preoperative); POST Th17 S: stimulated Th17-cells (absolute cell count/ μl) postoperative after clinical improvement; POST Th17 UN: unstimulated Th17-cells (absolute cell count/ μl) postoperative after clinical improvement; CO Th17 S: stimulated Th17-cells (absolute cell count/μl) at control examination after 6 months; CO Th17 UN: unstimulated Th17-cells (absolute cell count/μl) at control examination after 6 months; IL-17: Interleukin-17; μl: microliter; pg/ml: picogram/milliter; IVDH: Intervertebral Disc Herniation

| **PN** | **Breed** | **Age**  (in years) | **Sex** | **Weight** (kg) | **Th17 S**  (absolute cell count/μl) | **Th17 UN**  (absolute cell count/μl) | **IL-17 Serum** (pg/ml) | **IL-17 Liquor** (pg/ml) |
| --- | --- | --- | --- | --- | --- | --- | --- | --- |
| **101** | BA | 3 | M | 14.6 | 87.28 | 40.91 | 155.06 | 2.53 |
| **102** | BA | 1 | M | 10.7 | 51.72 | 20.14 | - | - |
| **103** | BA | 3 | M | 14.8 | 26.38 | 6.06 | - | - |
| **104** | BA | 3 | M | 14.5 | 26.88 | 2.45 | 78.75 | 8.76 |
| **105** | BA | 5 | M | 16.5 | 24.52 | 9.95 | 35.52 | 2.64 |
| **106** | BA | 1 | F | 13.4 | 31.62 | 16.2 | - | - |
| **107** | BA | 1 | F | 15.0 | 47.22 | 15 | 14.99 | 0.1 |
| **108** | BA | 1 | M | 12.0 | 49.57 | 14.98 | - | - |
| **109** | BA | 1 | M | 13.8 | 74.62 | 46.31 | - | - |
| **110** | BA | 1 | M | 13.2 | 56.1 | 32.09 | 72.93 | 2 |
| **111** | BA | 2 | M | 13.3 | - | - | 34.77 | 2.86 |
| **112** | BA | 4 | F | 11.7 | - | - | 63.16 | 3.04 |
| **113** | BA | 2 | F | 13.9 | - | - | 75.58 | 0.1 |
| **114** | BA | 2 | M | 10.2 | - | - | 105.58 | 0.1 |
| **115** | BA | 2 | F | 12.7 | - | - | 722.27 | 2.53 |

PN: patient number; BA: Beagle; M: male; F: female; kg: kilogram; Th17 S: stimulated Th17-cells (absolute cell count/ μl); Th17 UN: unstimulated Th17-cells (absolute cell count/ μl); IL-17: Interleukin-17; μl: microliter ;pg/ml: picogram/milliter
